# Scope actuation system for articulated laparoscopes

**DOI:** 10.1007/s00464-023-09904-z

**Published:** 2023-02-07

**Authors:** Nihal Abdurahiman, Mohammad Khorasani, Jhasketan Padhan, Victor M. Baez, Abdulla Al-Ansari, Panagiotis Tsiamyrtzis, Aaron T. Becker, Nikhil V. Navkar

**Affiliations:** 1 Department of Surgery, Hamad Medical Corporation, Doha, Qatar; 2 Department of Electrical Engineering, University of Houston, Houston, TX USA; 3 Department of Mechanical Engineering, Politecnico di Milano, Milan, Italy; 4 Department of Surgery, Surgical Research Section, Hamad General Hospital, Hamad Medical Corporation, PO Box 3050, Doha, Qatar

**Keywords:** Laparoscopic surgery, Robotic scope assistant system, Scope holders, Articulated scopes, Surgical robots

## Abstract

**Background:**

An articulated laparoscope comprises a rigid shaft with an articulated distal end to change the viewing direction. The articulation provides improved navigation of the operating field in confined spaces. Furthermore, incorporation of an actuation system tends to enhance the control of an articulated laparoscope.

**Methods:**

A preliminary prototype of a scope actuation system to maneuver an off-the-shelf articulated laparoscope (EndoCAMaleon by Karl Storz, Germany) was developed. A user study was conducted to evaluate this prototype for the surgical paradigm of video-assisted thoracic surgery. In the study, the subjects maneuvered an articulated scope under two modes of operation: (a) *actuated mode* where an operating surgeon maneuvers the scope using the developed prototype and (b) *manual mode* where a surgical assistant directly maneuvers the scope. The actuated mode was further assessed for multiple configurations based on the orientation of the articulated scope at the incision.

**Results:**

The data show the *actuated mode* scored better than the *manual mode* on all the measured performance parameters including (a) total duration to visualize a marked region, (a) duration for which scope focus shifts outside a predefined visualization region, and (c) number of times for which scope focus shifts outside a predefined visualization region. Among the different configurations tested using the actuated mode, no significant difference was observed.

**Conclusions:**

The proposed articulated scope actuation system facilitates better navigation of an operative field as compared to a human assistant. Secondly, irrespective of the orientation in which an articulated scope’s shaft is inserted through an incision, the proposed actuation system can navigate and visualize the operative field.

**Supplementary Information:**

The online version contains supplementary material available at 10.1007/s00464-023-09904-z.

An articulated scope comprises a rigid shaft with an articulated distal end to change the viewing direction. These scopes improve navigation of the operating field by: (a) Providing distal adjustment of the viewing direction in confined spaces (such as thoracic [1] or insufflated abdominal [2] cavity) without the need to move the scope’s shaft [3]; (b) Allowing a surgeon to look around the corner and even go over an anatomical structure to look behind it in a cavity [4]; and (c) Facilitating hand–eye coordination in para-axial setup [5]. In a para-axial setup (contrary to commonly used co-axial setup), the axis along the laparoscopic instruments is not aligned with the axis along the scope. This provides a wider range for positioning trocars to insert laparoscopic instruments, which in turn provides sufficient space to the surgeon for holding and moving the instruments while operating.

These advantages led to development of commercial articulated scopes (such as EndoEyeFlex—Olympus, EndoCAMeleon—Karl Storz, and Ideal Eyes—Stryker). In addition to the commercial products, several prototypes of robotic (or actuated) articulated scopes comprised a rigid shaft and an actuated flexible distal end have been proposed [3, 6, 7]. The Cardioscope system proposed by Li et al. [6] showed the feasibility of exploring and visualizing the heart through a single incision. The PliENT system developed by Legrand et al. [3] assisted in accessing the maxillary sinus through the nasal cavity. Similarly, the robotic flexible system developed by Song et al. [7] used an actuated articulated scope in cholecystectomy procedures. In these prototypes, the actuation mechanism is prebuilt into the robotic scope and causes the flexible distal end to bend and sweep a region. This design poses several challenges. First, the bending section may interfere with tissues as well as with laparoscopic instruments, especially in a complex confined space. Such interference may inadvertently damage the components of the bending mechanism [8]. Second, the stability of the operating field view might be reduced because of the flexible nature of the scope’s bending section [9]. Third, after the surgery, sterilizing the complete scope system would be difficult due to the presence of integrated electromechanical actuation components [10]. In such cases, a robust and practical robotic articulated scope system, which can alter the viewing direction without the need of a bending section and is easy to sterilize after surgery, would be ideal.

Working towards this direction, this paper presents a preliminary prototype of a scope actuation system that uses an off-the-shelf articulated scope (EndoCAMeleon, Karl Storz, Germany). The scope has a rigid shaft without a flexible bending section. The articulation is produced mechanically via a rotating prism mechanism at the distal end (i.e. without any integrated electromechanical components) [11]. The objective of this work is to evaluate the proposed scope actuation system using two criteria: (a) the capability of the system to navigate and visualize an operative field when the articulated scope’s shaft is inserted in different orientations through the incision, and (b) the performance of the system in maneuvering the articulated scope as compared to a human assistant.

## Materials and methods

### Scope actuation system

**Fig. 1:**
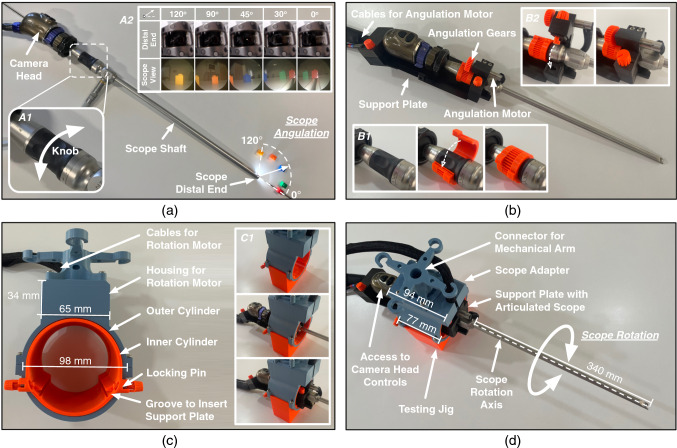
**a** An articulated scope (EndoCAMeleon by Karl Storz) connected to a camera head (Image1HD by Karl Storz). Rotating the knob at the rear end of the scope (shown in Panel A1) controls the scope angulation, rotating the viewing direction from 0° to 120° (shown in Panel A2). **b** The articulated scope and the camera head are placed on a support plate. A gear mechanism is attached to the knob (as shown in Panel B1), which is engaged by the angulation motor (shown in Panel B2). Actuating the angulation motor rotates the knob, which in turn changes the scope angulation. **c** A scope adapter is used to host the support plate. The support plate is inserted inside the inner cylinder (shown in Panel C1) along the groove and locked in position using the locking pins. **d** The scope adapter is equipped with a connector to attach the assembly to a mechanical arm. Rotation of the inner cylinder with respect to the outer cylinder rotates the articulated scope along its axis

The articulated scope is hosted on a surgical scope adapter (Fig. 1). The scope adapter has two Degrees-of-Freedom (DoF) for actuation. The first DoF controls the rotation of the scope along its shaft, while the second DoF manipulates the angulation of the scope’s viewing direction from 0° to 120°. During the surgery, the articulated scope along with the camera head is first placed on the scope adapter. Then the shaft of the articulated scope is inserted through an incision to view the operating field inside the cavity. A passive mechanical arm (affixed next to the operating table) is used to suspend the scope adapter. Details of the computer-aided designs, fabrication, and the measured technical performance of the surgical scope adapter prototype are presented in Khorasani et al. [12].

**Fig. 2:**
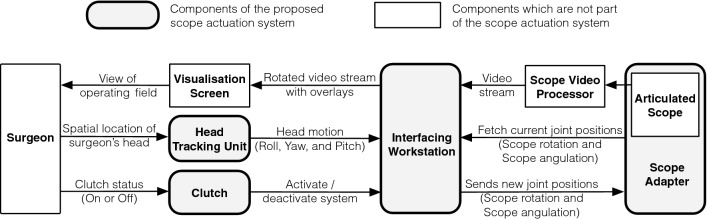
Architecture of the proposed system for actuating an articulated scope using the surgeon’s head motions. The hardware of the system includes a head tracking unit, a clutch, an interfacing workstation, and the scope adapter. The interfacing workstation acts as a computational unit to process the commands and data streams to/from different hardware units

**Fig. 3:**
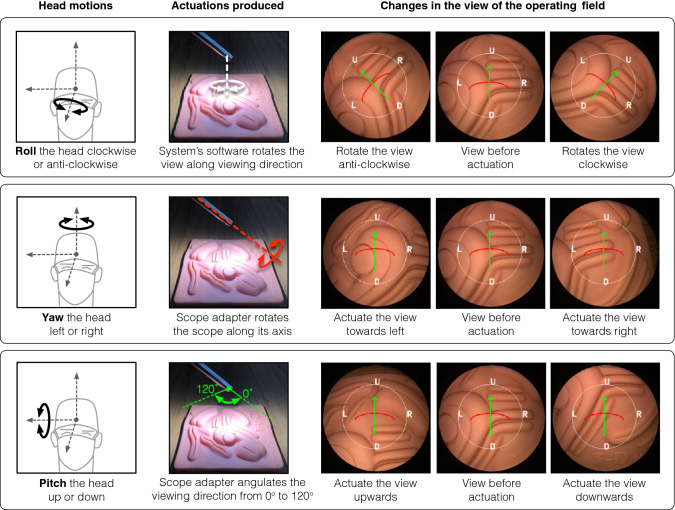
The three head motions (roll, yaw, and pitch) performed by the surgeon to interact with the system, the actuation produced by the system based on the perceived head motions, and changes in the view of the operating field based on the actuation produced by the system

The architecture of the system for actuating the articulated scope is presented in Fig. 2. A surgeon interacts with the system using head motions and a clutch. A clutch (controlled by a foot pedal) is used to activate/deactivate the system. When the clutch activates the system, the orientation of the surgeon’s head is tracked by the head tracking unit and decomposed into roll, yaw, and pitch rotations. The interfacing workstation processes these head motions and uses them to steer the view of the operating field (as shown in Fig. 3). It fetches the current joint positions (for scope rotation and scope angulation) from the scope adapter, increments or decrements the values based on the perceived head motions, and sends the newly computed joint positions back to the scope adapter for actuation. The pitch and yaw of the surgeon’s head are used for angulation and rotation of the articulated scope, respectively. The interfacing workstation also rotates the video stream acquired from the scope video processor based on the roll angle of the surgeon’s head. As the camera head and the scope rotate together, the software-enabled rotation ensures the horizon of the operative field is kept intact. The software rotation also enables the scope adapter (along with the connector) to be rotated along the scope’s shaft direction and placed in any desired position while connecting with the passive mechanical arm. The rotated video stream of the operative field is displayed on a visualization screen, which is then perceived by the surgeon.

### Experimental setup

#### Surgical scenario

**Fig. 4:**
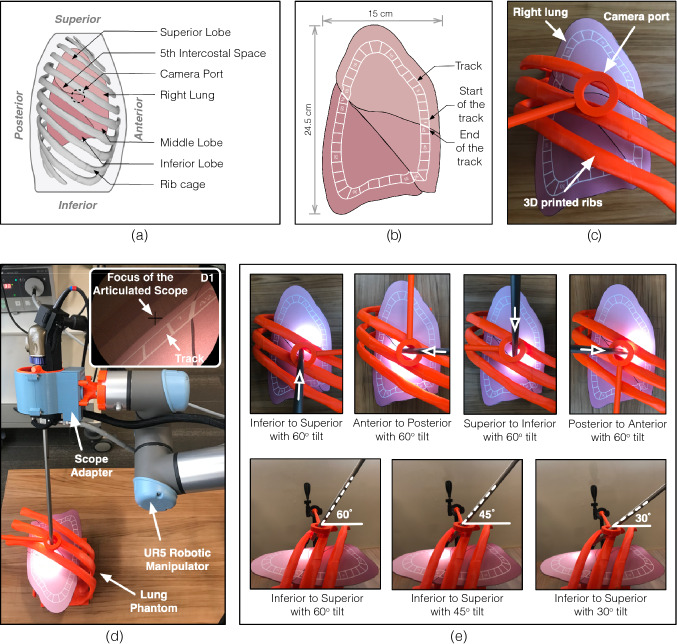
**a** Pictorial representation of a patient in left lateral decubitus position depicting right lung lobes and ribs. **b** A closed loop track drawn on the right lung lobes for the subject to visualize during the navigation task. **c** Fabricated lung phantom used in the study. **d** Scope adapted connected to a UR5 robotic manipulator for placing the articulated scope with respect to the lung phantom. The view acquired by the articulated scope is shown in Panel D1. **e** Configurations (representing the orientation of the articulated scope shaft) used in the study

A surgical paradigm of video-assisted thoracic surgery (VATS) was selected as it requires extensive navigation to visualize the complete thoracic cavity [13, 14]. A phantom representing the right lung of a patient in the left lateral decubitus position was fabricated (Fig. 4). The phantom depicted the right superior, middle, and inferior lobes along with the horizontal and oblique fissures. The phantom also included three ribs representing the fifth and sixth intercostal space. For the experiments, the articulated scope’s shaft was inserted through a circular ring to simulate an incision for the camera port.

#### Subjects

A user study was conducted with five subjects. The subjects were researchers from the department of surgery at Hamad General Hospital, Qatar with previous experience in maneuvering an articulated scope (to maintain horizon, ensure optimum distance from operative field, and keep the active view in the center of the screen). The study was approved by the institutional review board ethical committee (Medical Research Center, Doha, Qatar, approval number MRC-03-21-152).

#### User study

Before the user study, the subjects went through a 10–15 min preparatory session to gain familiarity with controlling the viewing direction using the head-motion based control of the scope actuation system. The preparatory session was completed after each subject was able to map the head motions to the individual actuations produced by the system (as depicted in Fig. 3). The subjects then took part in the user study and were asked to perform a task of maneuvering the articulated scope to visualize the peripheral regions of the lung phantom. A closed-loop track with blocks numbered sequentially from #1 to #42 was drawn on an outline of the lung that covered the three lobes (Fig. 4b). The subjects were asked to maneuver the articulated scope to visualize each block of the track starting from block #1 and ending at block #42 in an anticlockwise direction on the visualization screen. While maneuvering the articulated scope, each block should appear in the center of the visualization screen. This ensures the viewing direction is oriented towards the track. The rationale of using a closed-loop track is if an operator can traverse and view the peripheral regions, the operator would also be able to view the regions located in the center.

The scope was maneuvered under two modes of operation: *actuated* and *manual*. In the *actuated mode*, the subjects used the proposed scope actuation system in the six different configurations shown in Fig. 4e. The scope adapter was connected to a UR5e robotic manipulator (Universal Robots, Denmark) in the *actuated mode* (Fig. 4d). Each configuration was stored in the robotic manipulator to ensure repeatability across different subjects while performing the user study. In each configuration, the articulated scope shaft was inserted at a unique orientation through the incision (as shown in Fig. 4e). In the *manual mode*, the subjects were asked to maneuver the articulated scope manually without the scope actuation system. The model of the operating surgeon providing verbal commands to the assistant surgeon for maneuvering the scope during the surgery was not used as it would have introduced bias. The bias would be in form of additional errors due to the miscommunication between the two surgeons [15, 16].

#### Data collection

During the study, the video stream displayed on the visualization screen was recorded. The recorded video stream was processed manually by a research assistant from department of surgery at Hamad General Hospital, Qatar. The subjects and the modes of operation were pseudo-anonymized before giving the videos to the research assistant for processing. The following measures related to the functioning of the proposed scope actuation system were extracted: (a) duration to navigate the track by maneuvering the articulated scope, (b) duration for which the focus of the articulated scope shifts outside the track while navigating, and (c) an error count equal to the number of times the focus of the articulated scope shifts outside the track while navigating. The focus of the articulated scope is shown in Panel D1 of Fig. 4d as a “ + ” sign rendered in the center of the view acquired from the articulated scope.

Towards the end of the study, a NASA Task Load Index (TLX) scale was used to measure the perceived workload in maneuvering the scope during manual mode (with the subject behaving as an assistant to the operating surgeon) and actuation mode (with the subject behaving as the operating surgeon). The scale has been used in previous studies for the assessment of user experiences with surgical scope actuation systems [7, 17]. In the NASA-TLX scale, a set of subjective questions on a scale of 1 to 10 assess the mental demand, physical demand, temporal demand, effort, performance, and frustration level.

#### Data analysis

To examine whether each of the three measures (extracted from the recorded video stream) as response variables exhibit statistically significant differences among the seven levels of the explanatory variable ‘Mode’ (i.e., six for *actuated mode* and one for the *manual mode*), Mixed Effects Modeling (MEM) was used. Each of the five participating subjects performs the task with all possible modes, giving rise to a repeated measure design. Thus, using MEM we were able to examine the significance of the explanatory variable ‘Mode’ (that will play the role of the fixed effect) taking account the subject-to-subject variation (as the subjects were used as random effects). In the analysis, *manual mode* was used as a baseline and each of the six *actuated modes* was examined whether significant statistical differences exist on the mean response compared to the baseline. Finally, to examine whether the six *actuated modes* have any significant difference from each other, Tukey’s multiple comparison method was used, comparing all possible pairs.

## Results

**Fig. 5:**
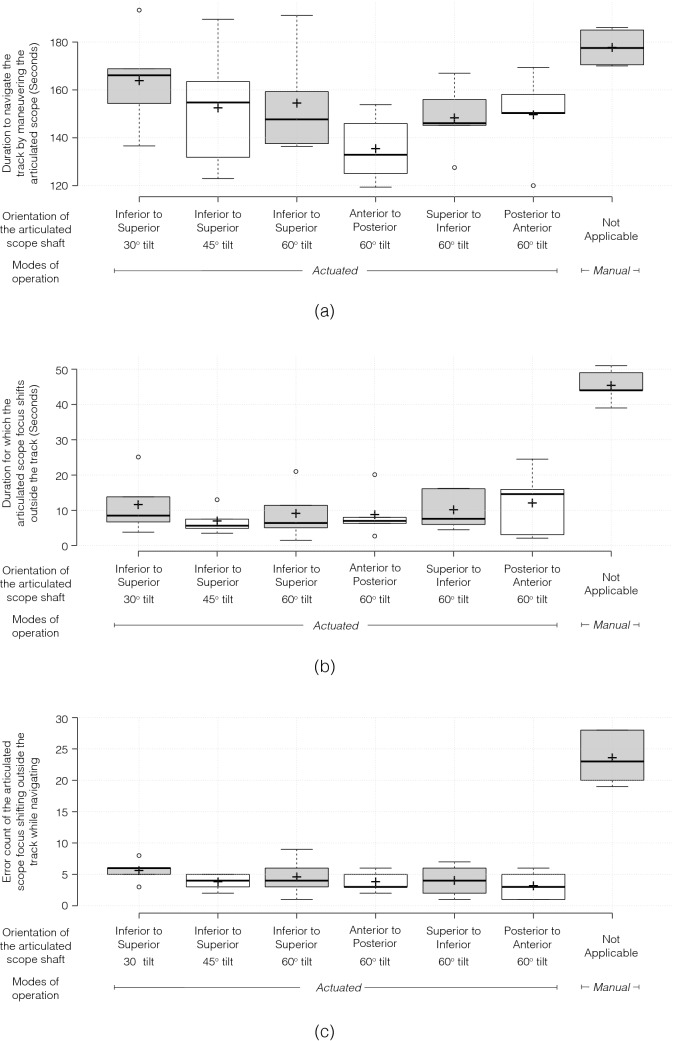
Boxplots presenting parameters used to assess the user-study task of navigating a track. The track was navigated using an articulated scope under manual and actuated mode of operations. The actuated mode was further categorized into six different configurations

The results of the user study are summarized in Fig. 5 (and the MEM output are described in the supporting document). The duration to navigate the track by maneuvering the articulated scope is presented in Fig. 5a. Participants took longer to navigate the track using the *manual mode* than they did using the *actuated mode*. The average durations are significantly reduced (*p* < 0.05*)* for the *actuated modes* compared to the *manual mode* configurations except for inferior to superior with 30° tilt. Figure 5b and Fig. 5c show the duration and the number of times (as an error count) for which the articulated scope focus shifts outside the tract. The average duration and the error count for *actuated modes* is significantly lower (*p* < 0.001) for all the six configurations as compared to the *manual mode*. Based on Tukey’s multiple comparison tests, no significant difference was found among the average time taken to navigate the track, and the average duration and the error count for which the articulated scope focus shifts outside the tract for the six configurations of the *actuated modes*.

**Fig. 6:**
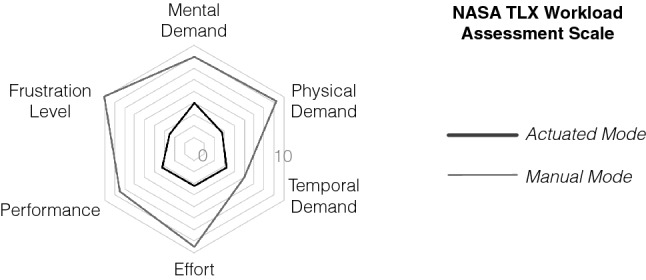
The average scores using the NASA-TXL workload assessment scale (from 1 to 10) for actuated mode and manual mode

The NASA-TLX scores using *actuated mode* verses *manual mode* are shown in Fig. 6. A lower score reflects a better evaluation for mental demand, physical demand, temporal demand, effort, and frustration level. In the case of performance, a lower score reflects it was good whereas a high score reflects it was poor. The *actuated mode* performs better as compared to *manual mode* on the NASA-TLX scale across all the subjective questions.

## Discussion

The data show that the *actuated mode* scored better than the *manual mode* on all the measured performance parameters, including duration to complete the task, error counts, and duration for which scope focus shifts outside the track, as well as on NASA-TLX workload assessment scale. This suggests the proposed system has the potential to outperform a human assistant in maneuvering the articulated scope to navigate an operative field. One reason is that altering the view of operating field manually on the visualization screen requires the subject to rotate three components of the scope located along a collinear axis. This includes: (a) rotation of the scope’s shaft for traversing the track, (b) rotation of the scope’s knob for articulation, and (c) rotation of the camera head for rotating the view on the visualization screen. Due to the collinearity of the three rotational axes, it was difficult for the subjects to simultaneously perform the rotation actions in the *manual mode*. This was resolved in *actuated mode* because the operator’s head acts as a three-axis gimbal. Each actuation required for rotating the scope is mapped to a unique head movement.

In *manual mode*, a human assistant holding an articulated scope during a surgery can control a limited number of buttons and rotation knobs. Though the additional degree of freedom tends to provide better visualization, it leads to a practical limitations due to increased complexity in maneuvering the scope [9]. In such scenarios, a robotic scope adapter would provide ergonomic control for actuating an articulated scope.

Another observation made during the study in the *manual mode* was that the subjects primarily panned and tilted the scope shaft instead of rotating the scope shaft and angulating the view. These are natural maneuvers which are performed predominantly by a surgical assistant while navigating an operative field using zero-degree or angulated scopes. However, if the same actions are performed to navigate and visualize the insufflated cavity using articulated scope (as simulated in the task), it may cause interference with the operating surgeon’s hand holding the laparoscopic instruments. The proposed scope actuation system overcomes this by eliminating the need for pan and tilt maneuvers by keeping the scope’s axis stationary and providing multidirectional stable views for navigation.

Furthermore, the data show no statistical difference among the recorded parameters for the six configurations under the *actuation mode*. This implies that the proposed actuation system can navigate and visualize the operative field irrespective of the orientation in which an articulated scope’s shaft is inserted through an incision. In a regular minimally invasive surgery the principle of triangulation is used [18] while placing the trocars for inserting the laparoscopic instruments and the scope. That means the axes from the operating surgeon to the visualization screen and from the laparoscope to the targeted tissue should be collinear to achieve proper hand–eye coordination [5]. However, such an ideal trocar’s position is not always possible during certain surgeries due to constraints imposed by the anatomy. Triangulated placement limits the workspace for movement of the instruments and the scope inside insufflated cavity. In such cases, the use of articulated scope and the scope actuation system gives the flexibility to insert and actuate the scope at a desired orientation.

The surgical scope adapter (hosting an articulated scope) can be operated while connected to either a passive mechanical arm or an actuated robotic manipulator. In the case of a passive mechanical arm, the position of the scope tip is stationary, and the viewing direction is altered by roll, yaw, and pitch motion of the head. This is suitable for surgical scenarios where the scope’s shaft motion is restricted [13, 14] or remains stationary for most of the procedure [7, 19]. On the other hand, in the case of a robotic manipulator, the position of the scope tip can be changed by maneuvering the scope (hosted on the scope adapter). This would be suitable for surgical scenarios which require frequent maneuvers of the scope’s shaft [6, 20]. In such scenarios, a robotic manipulator with at least five degrees-of-freedom would be required to position and orient the scope adapter and would enable scope movements comprising insertion/retraction, panning left/pan right, and tilting up/tilt down, while maintaining a remote center of motion at the incision point. It would also require modifying the interface to incorporate three additional DoF for translation of the scope tip along with the current three DoF to alter the viewing direction (currently based on roll, yaw, and pitch motion of the head). The movements of the scope tip can be achieved by: (i) using the same interface and converting the translation motion of the head (along X, Y, and Z directions) to actuation commands for robotic manipulator (such as in [21]), or (ii) using a separate interface (such as a miniature joystick attached to the laparoscopic instrument [22], or voice control commands [23]). The former interface would be intuitive if there is one-to-one mapping between the movement of the surgeon’s head to motion of the virtual camera at the scope tip [24]. The latter interface would require the articulated scope angulation to be set near to zero-degree while moving the scope tip. It will ensure nearby tissue structures are visible while moving the scope to avoid impingement. In addition, holographic technologies (that render a mixed reality environment [25, 26]) can also be used to project holograms of the scope shaft and the scope’s viewing frustum onto the patient’s body, producing a see-through effect [21].

The study has certain limitations. First, during VATS, one-lung ventilation is commonly used that causes one of the lungs to deflate and collapse [27]. During the study, a non-deflated model was shown to the subject. The rationale was to demonstrate the visualization capability of the proposed system inside the thoracic cavity surrounded by rib cage. The non-deflated model of lung represented the boundaries of the thoracic cavity. Secondly, this was a preliminary user study with a low sample size (*n* = 5) to assess the potential of developed scope actuation system for actuation of an articulated scope. Further randomized control studies with increased sample size and varying levels of surgical expertise would be required to assess the user interfacing [28–30], hardware components, and eventually demonstrate the advantages of the system in different surgical scenarios. As a part of future work, we plan to (a) design new support plates to host other articulated scopes (such as EndoEye Flex LTF—Olympus and Ideal Eyes—Stryker), and (b) assess the usage of such scope adapters for procedures (such as colectomies [19] and cholecystectomy [7]) that require localized visualization of the operating field.

## Supplementary Information

Below is the link to the electronic supplementary material.

Supplementary file1 (PDF 56 kb)
