# Supplementary material for: Scope actuation system for articulated laparoscopes
Source: Surg Endosc. 2023 Feb 7;37(3):2404–13. doi: 10.1007/s00464-023-09904-z (PMC10017632; doi:10.1007/s00464-023-09904-z)
Supplement: Supplementary file 1 — Supplementary file1 (PDF 56 kb) [file 464_2023_9904_MOESM1_ESM.pdf]

Supporting Document

In the mixed effects model for all three response variables the Manual Mode was treated as baseline and the mixed effect model examined whether each of the remaining six modes have significant differences when compared with the baseline. The model outputs per response are as follow:

Duration to complete the task

```
Fixed effects:
                                Estimate Std. Error   df t value Pr(>|t|)    
(Intercept)                     178.16       9.37  24.08  19.014 5.24e-16 ***
Inferior_to_Superior_30_Tilt    -14.32      11.43  23.41  -1.253  0.22265    
Inferior_to_Superior_45_Tilt    -25.65      11.43  23.41  -2.244  0.03455 *  
Inferior_to_Superior_60_Tilt    -23.75      11.43  23.41  -2.078  0.04886 *  
Anterior_to_Posterior_60_Tilt   -42.70      11.43  23.41  -3.737  0.00105 ** 
Superior_to_Inferior_60_Tilt    -29.79      11.43  23.41  -2.607  0.01564 *  
Posterior_to_Anterior_60_Tilt   -28.56      11.43  23.41  -2.500  0.01987 *  
```

Duration for which scope focus shifts outside the track

```
Fixed effects:
                                Estimate Std. Error   df  t value Pr(>|t|)    
(Intercept)                      45.400      3.061   9.582   14.83 6.29e-08 ***
Inferior_to_Superior_30_Tilt    -33.820      2.852  24.000  -11.86 1.60e-11 ***
Inferior_to_Superior_45_Tilt    -38.492      2.852  24.000  -13.50 1.06e-12 ***
Inferior_to_Superior_60_Tilt    -36.330      2.852  24.000  -12.74 3.59e-12 ***
Anterior_to_Posterior_60_Tilt   -36.580      2.852  24.000  -12.83 3.11e-12 ***
Superior_to_Inferior_60_Tilt    -35.320      2.852  24.000  -12.38 6.49e-12 ***
Posterior_to_Anterior_60_Tilt   -33.360      2.852  24.000  -11.70 2.12e-11 ***
```

Error Counts

```
Fixed effects:
                              Estimate Std. Error      df t value Pr(>|t|)    
(Intercept)                     23.600      1.159   9.930   20.37 1.99e-09 ***
Inferior_to_Superior_30_Tilt   -18.000      1.098  24.000  -16.39 1.56e-14 ***
Inferior_to_Superior_45_Tilt   -19.800      1.098  24.000  -18.02 1.87e-15 ***
Inferior_to_Superior_60_Tilt   -19.000      1.098  24.000  -17.30 4.70e-15 ***
Anterior_to_Posterior_60_Tilt  -19.800      1.098  24.000  -18.02 1.87e-15 ***
Superior_to_Inferior_60_Tilt   -19.600      1.098  24.000  -17.84 2.35e-15 ***
Posterior_to_Anterior_60_Tilt  -20.400      1.098  24.000  -18.57 9.56e-16 ***
```

```
---
Significance codes:  0 ‘***’ 0.001 ‘**’ 0.01 ‘*’ 0.05 ‘.’ 0.1 ‘ ’ 1
```
